# Fibrotic Remodeling of the Extracellular Matrix through a Novel (Engineered, Dual-Function) Antibody Reactive to a Cryptic Epitope on the N-Terminal 30 kDa Fragment of Fibronectin

**DOI:** 10.1371/journal.pone.0069343

**Published:** 2013-07-23

**Authors:** Maryada Sharma, Anil Tiwari, Shweta Sharma, Preeti Bhoria, Vishali Gupta, Amod Gupta, Manni Luthra-Guptasarma

**Affiliations:** 1 Department of Immunopathology,Postgraduate Institute of Medical Education and Research, Chandigarh, India; 2 Department of Internal Medicine,Postgraduate Institute of Medical Education and Research, Chandigarh, India; 3 Department of Ophthalmology,Postgraduate Institute of Medical Education and Research, Chandigarh, India; University of California San Francisco, United States of America

## Abstract

Fibrosis is characterized by excessive accumulation of scar tissue as a result of exaggerated deposition of extracellular matrix (ECM), leading to tissue contraction and impaired function of the organ. Fibronectin (Fn) is an essential component of the ECM, and plays an important role in fibrosis. One such fibrotic pathology is that of proliferative vitreoretinopathy (PVR), a sight-threatening complication which develops as a consequence of failure of surgical repair of retinal detachment. Such patients often require repeated surgeries for retinal re-attachment; therefore, a preventive measure for PVR is of utmost importance. The contractile membranes formed in PVR, are composed of various cell types including the retinal pigment epithelial cells (RPE); fibronectin is an important constituent of the ECM surrounding these cells. Together with the vitreous, fibronectin creates microenvironments in which RPE cells proliferate. We have successfully developed a dual-action, fully human, fibronectin-specific single chain variable fragment antibody (scFv) termed Fn52RGDS, which acts in two ways: i) binds to cryptic sites in fibronectin, and thereby prevents its self polymerization/fibrillogenesis, and ii) interacts with the cell surface receptors, ie., integrins (through an attached “RGD” sequence tag), and thereby blocks the downstream cell signaling events. We demonstrate the ability of this antibody to effectively reduce some of the hallmark features of fibrosis - migration, adhesion, fibronectin polymerization, matrix metalloprotease (MMP) expression, as well as reduction of collagen gel contraction (a model of fibrotic tissue remodeling). The data suggests that the antibody can be used as a rational, novel anti-fibrotic candidate.

## Introduction

Persistent stimulus of chronic inflammation, in response to infections, autoimmune reactions, trauma, and other types of tissue injury, can result in fibrosis, which is characterized by excessive deposition of extracellular matrix (ECM) components. Fibronectin (Fn) matrix assembly is a major contributing factor to the switch from normal tissue repair to a fibroproliferative response. Such an aberrant wound healing mechanism has been related to several pathologies [1]. Proliferative vitreoretinopathy (PVR) is a fibrotic disorder of the eye, resulting from a failure of surgical repair of rhegmatogenous retinal detachment. Following breakdown of the blood-retinal barrier, plasma fibronectin gains entry into the subretinal space, and acts as a chemo attractant, causing migration of the RPE cells out into the vitreous [2], [3]. The vitreous provides a conducive microenvironment for the RPE cells to proliferate, which in turn synthesize excessive ECM. This ECM on the side of the vitreous is called the epiretinal membrane, while the ECM formed between the RPE cells and the photoreceptors is called the subretinal membrane. Both these membranes are rich in RPE cells, and can contract and pull onto the retina. The pathology in PVR is thus considered to be an exaggerated wound-healing response by the retinal pigment epithelial cells, involving inflammation, extracellular matrix deposition and tissue remodeling.

Fibronectin plays a particularly important role in the fibrotic pathology [4], because it takes part in i) cell-cell and cell-substratum adhesion [5]; ii) assembly of other components of the ECM such as collagen types I and III, which depend on the formation and availability of pre-formed fibronectin matrix [6]; and iii) adhesion-dependent cell growth [7] and cell contractility [8].

**Figure 1:**
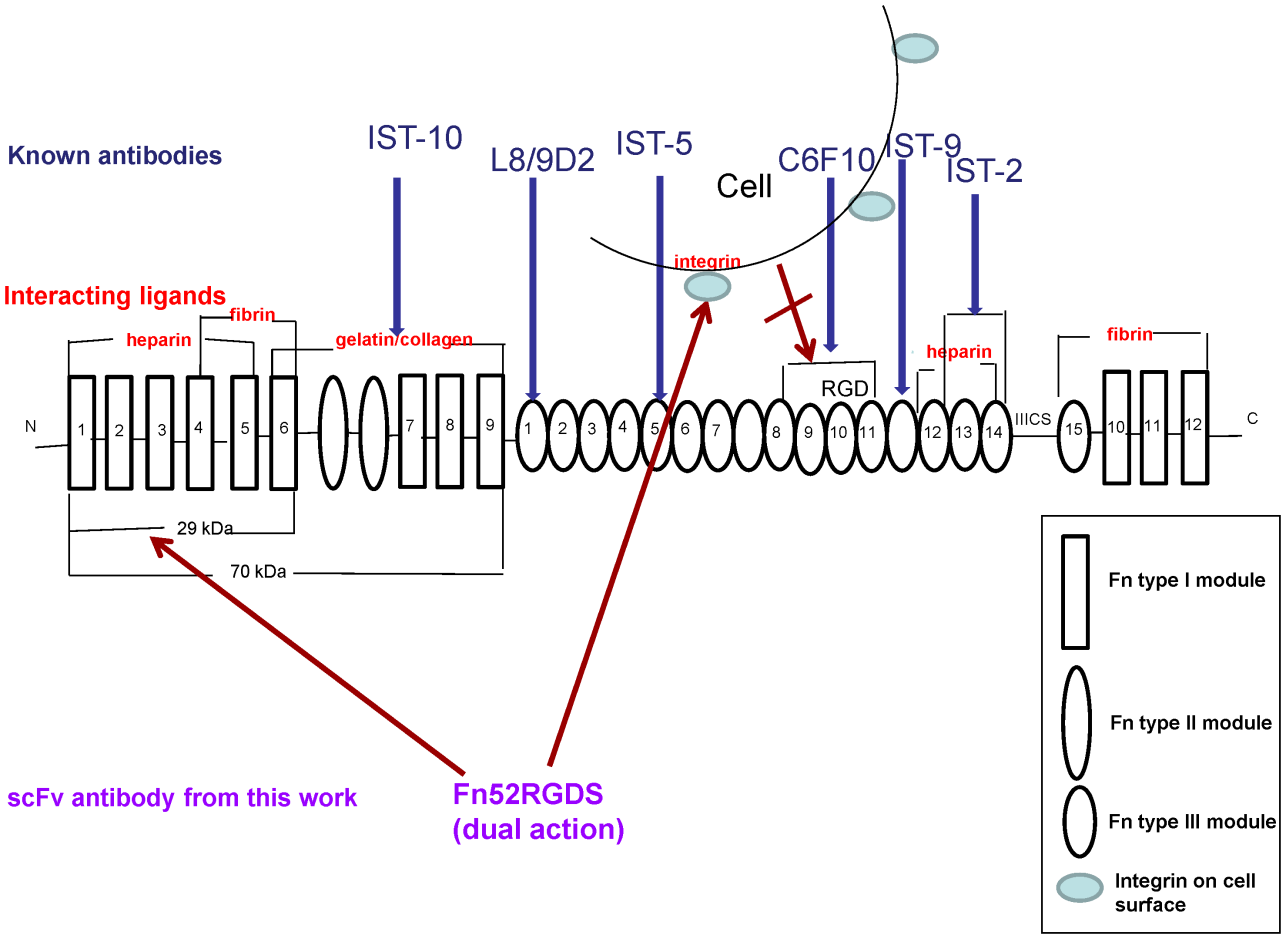
A schematic diagram to illustrate the modular structure of fibronectin, sites of interaction of some of the known anti-fibronectin antibodies, and the proposed dual action of the scFv antibody, Fn52RGDS. The double arrow indicates the interaction of the cell surface integrin with the “RGD” residues of fibronectin.

Fibronectin exists in two forms: the soluble form circulates in the plasma, while the insoluble form exists within the extracellular matrix as insoluble fibrils, subsequent to polymerization of the soluble form. The formation of the insoluble fibrillar networks of fibronectin in the ECM is a tightly regulated, step-wise process, initiated by the binding of fibronectin to cell surface receptors, via the 70 kDa N-terminal region of fibronectin [9], thereby triggering a signaling cascade and resulting in cytoskeletal remodeling through polymerization of actin fibers, and causing conformational changes within the fibronectin itself [9]–[11]. This results in the exposure of the ordinarily cryptic sites within the fibronectin’s type III domains [10], [11]. Exposure of these cryptic sites leads to i) interaction of this region with the 30 kDa N-terminal region of other fibronectin molecules, which causes the self association or polymerization of fibronectin [12] and, ii) engagement of the RGD residues within the fibronectin type III domain, with the α5β1 integrins on the cell surface, thus exposing matricryptic self-assembly sites within the fibronectin, aiding in self polymerization, as well as organization of the actin cytoskeleton to promote cell contractility [13], [14]. The exposure of additional binding sites thus helps in fibril formation, and the conversion of fibrils into an insoluble form [15]. There are various antibodies that have been developed to target either the sites involved in the self-polymerization of fibronectin, or sites on fibronectin which interact with the cell surface receptors (integrins), and these are shown in Figure 1.

The levels of fibronectin are high in both the membranes (subretinal and the epiretinal membrane), because of a) locally produced fibronectin (by the displaced retinal pigment epithelial cells), and because ofb) entry of plasma through the blood-retinal barrier [16]. Since fibronectin is a major player in the pathogenesis of PVR,we decided to use antibody engineering methods to develop an antibody capable of modulating the polymerization of fibronectin. The currently known antibodies (as shown in Figure 1) target individual sites on the fibronectin molecule; however, since alternative mechanisms exist for fibronectin fibrillogenesis (because of the multiple sites involved in self-polymerization), we designed an antibody which could possibly inhibit this assembly process by targeting two kinds of interactions – the Fn-Fn interaction, as well as the interaction of cell surface integrins with the Fn molecule.

We report that an scFv (single chain variable fragment) antibody, selected through phage display antibody library screening methods, against the N-terminal 30-kDa fragment of fibronectin, was further re-engineered to include an RGDS site (since the RGD sequence enables binding to integrins on cell surfaces) with an aim to make the scFv double-pronged in its action, i.e., act on both fibronectin, as well as cell surface integrins (Figure 1). Our data shows that Fn52RGDS does indeed act in both ways. Both antibodies were able to inhibit some of the key features involved in the PVR pathology, such as adhesion of RPE cells, and collagen gel contraction (which simulates the contraction of vitreous in human PVR); the re-engineered scFv (Fn52RGDS) is better than the parent molecule (Fn52) in terms of reducing fibronectin polymerization, migration of retinal pigment epithelial cells, and expression of matrix metalloproteases (MMP).

## Materials and Methods

### Ethics Statement

The vitreous and subretinal fluid samples were obtained for research use with the written consent of all the participants enrolled in a protocol, approved by the Institutional Ethics Committee of PGIMER; the guidelines of the Declaration of Helsinki were observed.

### Maintenance and Culture of Cell Lines

All cell lines used in the study are human derived.The retinal pigment epithelial cells (D407 or ARPE-19), and HT-1080 fibrosarcoma cells were maintained in DMEM media plus 10% FCS, supplemented with 100 U/ml penicillin, 100 mg/ml streptomycin, at 37°C and 5% CO_2_. The cells were used each time, after 2–3 passages.

### Preparation of Patient Vitreous Samples

Patients with phakic/pseudophakic retinal detachments undergoing pars plana vitrectomy were included in the study. In the beginning of the core vitrectomy, the assistant collected the cut vitreous using a tuberculin syringe. The subretinal fluid was collected using a soft-tip aspirating needle at the time of fluid gas exchange, and mixed with the vitreous sample, followed by dilution with DMEM media at a ratio of 1∶5 (vitreous:DMEM). This diluted sample preparation is referred to as “PV” throughout the text. The samples were then centrifuged at 12,000 g for 5 minutes at room temperature, divided into aliquots and stored at −80°C.

### Culture of D407 RPE/ARPE-19 Cells in the Presence of Patient Vitreous

RPE cells (seeding density of 50,000 cells per well) were seeded in a 24-well plate, with the PV, and various constructs of the scFv protein, in 500 µl total volume per well, and kept at 37°C and 5% CO_2_ for 24 h.

### Generation of Anti-fibronectin Antibody

Phage-display technology [17]was employed for generating antibody specific to the N terminal 30-kDa region of fibronectin (Sigma; F9911), using the human antibody scFv library (available as Tomlinson I and J, procured from MRC, UK). Three rounds of selection through biopanning were performed on the biotinylated N-terminal 30-kDa fibronectin fragment. The final round of biopanning was carried out using non-biotinylated fibronectin fragment as the target, in order to get rid of any contaminating streptavidin-binding phages. An aliquot of phage obtained as eluate from the fourth biopanning round was infected into exponentially growing HB2151 *E. coli* cells to produce soluble scFv, and 10-fold dilutions were grown on TYE plates containing ampicillin and 1% glucose.

### Purification of Soluble scFvFn52

The phagemid was transformed into *E.coli* HB2151 strain (a non-suppressor strain), and the cells were grown overnight to produce soluble scFv as described earlier [18]. Both the scFv antibodies were freshly dialyzed against PBS, prior to seeding of cells for the various experiments.

### Generation and Purification of an Irrelevant (Unrelated) scFv

Similar protocol as described above was used independently to generate an scFv against thrombospondin, to serve as a negative control in the study. This scFv was labeled O27.

### Cell-ELISA Assay

D407 RPE cells (100 µl of a suspension of 2×10^6^ cells/ml) were added to poly-L-lysine-coated wells in a 96-well plate or alternatively these cells were grown to confluence in a non-poly-L-lysine-coated 96-well plate and due to their adherent nature were directly used in ELISA. After three washings with 1× PBS, cells were fixed using absolute methanol for 5 min at room temperature. Internal peroxidases were inactivated by treatment with 0.3% hydrogen peroxide in absolute methanol for 30 min. This was followed by 3 washings with 1× PBS; wells containing cells were blocked with 5% BSA in 1× PBS overnight at 4°C. Primary antibody (scFv) was added into wells after washing with 1× PBS, and incubated for 2 h at room temperature. Mouse anti-His monoclonal antibody at a dilution of 1∶3000 was added to each well and incubated for 2 h. This was followed by incubation with anti-mouse HRP-conjugated antibody (1∶3000 dilution) for 1 h at room temperature. Color was developed with TMB; the reaction was stopped with 1N sulfuric acid, and the plates were read at 450 nm.

### Western Blot

Commercial fibronectin (Sigma Cat No. 2006) was probed on western blots using both, the scFv (periplasmic extract) as well as anti-Fn antibody (Sigma; 1∶400 dilution). In the case of the scFv, 1∶3000 dilution of mouse anti-His monoclonal antibody (Sigma, USA), followed by 1∶3000 dilution of anti-mouse HRP conjugated secondary antibody was used. For the anti-Fn antibody, anti-rabbit HRP conjugated secondary antibody (Sigma; 1∶3000) was used. The membranewas washed five times with TBS-Tween at room temperature and stained with HRP staining solution (DAB) till the signal was clearly visible (5–10 min). The reaction was stopped by rinsing two times in water.

### Genetic Manipulations

The re-engineering of the Fn52 clone to incorporate the codons corresponding to the RGDS sequence is schematically represented in Figure S1 in File S1. The scFvFn52 phagemid DNA was prepared and PCR was carried out to amplify the scFv fragment using the following set of primers:

forward primer (including the RGDS codons shown in bold, and an *NcoI* restriction site): 5′CAGCCGGCCATGGCC**AGAGGAGACTCG**GAGGTGCAGCTGTTGGAG 3′.

reverse primer (containing *NotI* restriction site) :

5′GATGATGTGCGGCCGCCCGTTTGATTTCC3’.

The conditions for PCR were as follows: initial denaturation for 5 min at 94^ο^C, followed by 30 cycles of 1 min denaturation at 94^ο^C, 1 min annealing at 55^ο^C, 2 min extension at 72^ο^C, and a further 10 min of extension at 72^ο^C. The amplified fragment (900 bp) was digested with NcoI and NotI respectively, and cloned into the phagemid vector pIT2. The transformed XL1Blue cells were used to prepare the scFvFn52RGDS DNA, which was sequenced to confirm the correctness of the frame of incorporation; this construct was labeled as scFv Fn52RGDS. The phagemid scFvFn52RGDS was transformed into HB2151 *E. coli* cells to produce soluble scFv using the same protocol as described above for the scFvFn52.

### Preparation of Fibronectin from Human Plasma

Fibronectin was purified from human plasma by a previously described protocol [19]. Briefly, nitrocellulose membrane was coated with 1% melted gelatin for 1 h at 37°C in a humid chamber. The membrane was washed 4 times with PBS, followed by fixation with 4% paraformaldehyde for 1 h, and incubated with diluted plasma for 1 h. After washing with PBS (4–5 times), it was incubated with 1 M NaCl at room temperature for 15 min. The bound fibronectin was eluted using freshly prepared 8 M urea in PBS. Before use, the fibronectin sample was concentrated and refolded by dialysis using progressively reduced concentrations of urea; the final dialysis was done using PBS.

### Immunofluorescence

ARPE-19 RPE cells were trypsinized and seeded in the presence of DMEM+patient-derived vitreous/subretinal fluid (PV) for 24 h at 37°C and 5% CO_2_, to allow fibronectin polymerization. Media was removed from the wells, washed with PBSfor 5 minutes, followed by fixation using 4% paraformaldehyde, and permeabilization with 0.5% Triton X-100. Blocking was done with 5% BSA (bovine serum albumin) for 1 h. The cells were incubated with primary antibody in BSA (1%) overnight, and FITC-labeled secondary antibody was used; images were captured on an Olympus confocal laser scanning microscope.

### Cell Viability Assay

The quantitation of cell viability was performed using the MTT assay kit (Millipore), using manufacturer’s instructions. Briefly, 10,000 cells were seeded and cultured in a 96-well plate in the presence of vitreous/SRF along with different concentrations of scFv, for 4 h at 37°C and 5%CO_2_. Controls were also set up to evaluate the effect of PBS on cells. MTT (3-(4, 5-dimethylthiazol-2-yl)-2, 5-diphenyltetrazoliumbromide); stock, 5 mg/ml) was added to a final concentration of 10% of the total volume in the well, and incubated overnight. Formazan crystals formed were dissolved in 100 ul of DMSO per well, and O.D was taken at 570 nm and reference at 630 nm. The optical density of the well containing cells seeded in the presence of patient vitreous/SRF was assigned as 100%.

### Cell Proliferation Assay

Cell proliferation was measured using the BrdU intake by flow cytometry. RPE cells were seeded at a density of 70,000 cells/well of a 24-well plate, in the presence of vitreous/SRF sample (diluted 5 times with DMEM), and cells were cultured for 36 h at 37°C and 5%CO_2_. The conditioned media from these wells was used for the cell proliferation assay. Briefly, 70,000 cells were seeded per well of a 24-well plate in the above conditioned media, along with the different scFv antibodies for 24 h at 37°C and 5%CO_2_, followed by addition of BrdU for 4–5 h. Following fixation and denaturation, BrdU labeling was detected using a mouse anti-BrdU antibody (1∶1000), followed by an FITC-conjugated anti-mouse antibody (1∶200). After washing with PBS, cells were analyzed by flow cytometry. Data was plotted as percentage inhibition of proliferation as compared to control (cells in the presence of patient vitreous/SRF alone), which was assigned a value of 100%.

### Cell Adhesion Assay

scFv antibodies (10–25 µg/ml) were coated on ELISA plate overnight in 0.1 M NaHCO_3_. The wells were washed with PBS two times, followed by blocking with 1% BSA for 1 h. Separately, cells were trypsinized, washed two times with serum-free media, and seeded at a density of 20,000 cells/well, in the presence of serum-free DMEM media, and incubated for 4 h at 37°C and 5%CO_2_. Media was discarded and wells were washed with PBS, followed by incubation in crystal violet stain (0.1% crystal violet in 10% ethanol) for 20 min. After gentle washing of wells with PBS, the dye was extracted using extraction buffer (0.2 M NaH_2_PO_4_ diluted in absolute ethanol (1∶1)), and absorbance read at 570 nm. Wells with no scFv coating (where only blocking was done with BSA) were used as a negative control. Fibronectin coating (25 µg/ml) was also done in some wells to serve as a positive control.

### Cell Migration Assay

Briefly, 2.5×10^5^ cells/ml were seeded onto the insert placed in a 24-well format in a total volume of 300 µl of serum-free DMEM media. The lower chamber contained the chemoattractant (fibronectin, 0.02 µM, purified from human plasma or a sample of patient-derived vitreous).This was used as a positive control. The other wells contained varying concentrations of the scFv in addition to fibronectin or vitreous. The transwell plates were incubated for 24 h at 37°C and 5% CO_2_. Cells that migrated through the 8 µm pore membranes, located at the bottom of every well insert, were stained and eluted. The optical density of the stained cells was then read by a colorimetric plate reader at 560 nm. Data are averages ± standard deviation of three experiments performed.

Migration experiments were also performed by pre-incubating the cells for 1 h with either i) scFv Fn52 (25 µg/ml), or ii) scFv Fn52 RGDS (25 µg/ml), or iii) varying concentrations of RGDS peptide (Sigma Chemicals; 0.5–5 µg/ml), or iv) varying concentrations of individual antibodies against alpha and beta integrins (anti-human integrin alpha 5; MAB1956Z; anti-human integrin beta 1; MAB1959; 0.1–1.0 µg/ml),or v) an irrelevant antibody (human IgG; Sigma Chemicals; 3.0 µg/ml), or vi) an antibody against fibronectin (Sigma Chemicals; F3648; 3.0 µg/ml). Following the incubation of the cells with each of the above reagents, migration was done in the same way as described above, with the lower chamber containing the chemoattractant, fibronectin in the presence of serum-free media.

### Assessment of Fibronectin Assembly

*ARPE-19,* retinal pigment epithelial cells were used for this assay, since these cells synthesize appreciable amounts of fibronectin in the presence of patient-derived vitreous/subretinal fluid (PV), unlike the D407 RPE cells. Cells were seeded at a density of 1×10^6^ cells/well in a 6-well plate, along with PV (prepared by dilution with DMEM media at a ratio of 1∶5 (vitreous:DMEM))and incubated for 24 h in a CO_2_ incubator, in the presence or absence of scFv antibodies. The supernatant was removed after 24 h, and cells were lysed with DOC lysis buffer as described earlier [20]. The DOC- soluble and insoluble fractions were separated by centrifugation, at 14,000 rpm for 30 min. The DOC-insoluble fraction was solubilized with the addition of 2% SDS, and the extent of fibronectin assembly into high molecular weight species was estimated [20], by 7.5% SDS–PAGE under reducing conditions. The proteins were transferred to nitrocellulose membrane, followed by immunoblotting using anti-fibronectin antibody (1∶5000; Sigma). The blots were developed by chemiluminescence (Amersham Biosciences) and exposed to X-ray film. Densitometric quantitation was performed using the Image-J software. In order to ensure that equal loading was done for all wells, the DOC-soluble fraction was similarly separated on 10% SDS-PAGE, followed by immunoblotting using mouse anti-GAPDH antibody (0411) (1∶5000, Santacruz Biotechnology, Inc) as internal control.

### Measurement of MMP Activity

ARPE-19 retinal pigment epithelial cells were seeded at a density of 1×10^5^ in a 24-well plate, along with PV, in the presence or absence of scFv antibodies for 24 h at 37°C and 5%CO_2_. The supernatant was centrifuged to remove any debris, and used for assessment of gelatinolytic activity [21]. To ensure that the cell numbers were comparable in all the wells, cells in each well were trypsinized and counted, after harvesting the CM. Accordingly, load volumes of CM were calculated to adjust for subtle differences in cell numbers. The experiment was conducted with four different patient-derived samples.

### Collagen Gel Contraction

A 24-well plate was coated with 400 ul of BSA (5 mg/ml in PBS) overnight.200 ul of BSA was removed per well, before adding cell-collagen mix to each well [cell collagen mix: 400 ul collagen solution (2 mg/ml) per well consisting of 0.5×10^6 ^cells]. The cells used for this assay were HT-1080 fibroblast cell line, since fibrosarcoma cells cause efficient contraction of collagen gels. The negative control included DMEM media; positive control was DMEM plus 0.5%FBS. Collagen was allowed to solidify for 90 min.Gel was carefully detached from along the walls using a syringe needle and media containing scFv antibodies (25 µg/ml, in the presence of 0.5% FBS.) was overlaid on the collagen gel. Area of the contracted gel was monitored after 4 h, and calculated using Image-J digital image analysis software, represented as gel to well ratio in percentage. Area of the blank well was taken be 100%, and data was plotted as percentage of gel to well ratio All experiments were performed as triplicate.

### Statistical Analysis

All data are expressed as means ± S.E.M. unless otherwise stated. Statistical analysis was performed by the one way Anova test. A *p* value less than 0.05 was considered to be significant.

## Results

### Generation and Characterization of an Antibody against the 30-kDa Fragment of Fibronectin

Phage display antibody library screening method was used to obtain scFv (single chain variable fragment) antibody specific to the 30-kDa N-terminal region of fibronectin. Four rounds of biopanning were performed to enrich the population of Fn-binding phages. Table S1 in File S1 shows the gradual increase in the number of output phages at each round, demonstrating the subsequent enrichment of fibronectin-binding phages at each step. The slight deviation in the output phage number in the fourth round of biopanning, may be attributed to the fact that this round of biopanning was carried out against non-biotinylated fibronectin, which may have resulted in elimination of all the biotin-binding phages present in the phage population. An aliquot of phage obtained as eluate from the fourth biopanning round was infected into exponentially growing HB2151 *E. coli* cells (a non-suppressor strain); the soluble antibodies produced in this strain, were screened for their binding ability to cell line-derived (D407) retinal pigment epithelial (RPE) cells, by Cell-ELISA. It is to be noted that although for the purpose of biopanning, the 30 kDa fragment of fibronectin was used, for the purpose of screening, we used cell-ELISA against RPE cells and from among the good binders obtained at this step, we evaluated these scFv clones for their binding ability to retinal tissue, as well as to lymphocytes(to exclude all possible non-specific clones). Finally, out of a total of 96 clones that were screened, one (Fn52) reproducibly showed good binding only to the RPE cells (Figure S2 in File S1), and not to either retinal tissue or lymphocytes.

**Figure 2:**
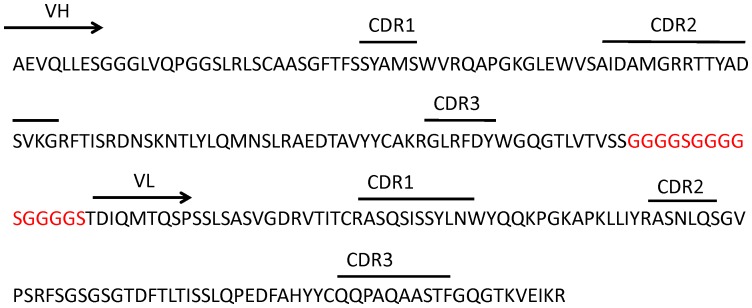
Amino acid sequence of the clone Fn52, showing the sequence of heavy and light chains, along with the CDR regions. The region in red shows the linker region between the heavy and light chains.

Sequence analysis of scFv Fn52 is represented in Figure 2. The heavy chain V segment belongs to the IGHV3 family, while the light chain V segment belongs to the VGKV1 subgroup.

A preparation of the periplasmic extract from this phagemid-containing clone was carried out, followed by purification on Ni-NTAgarose column. Figure S3A in File S1shows the SDS-PAGE of the eluted protein, and the corresponding western blot (using anti-His antibody).

The re-engineered scFv, Fn52RGDS was also purified likewise, and Figure S3B in File S1 shows the purified protein.

### Fn52 Binds to Cryptic Regions in the Fibronectin

**Figure 3:**
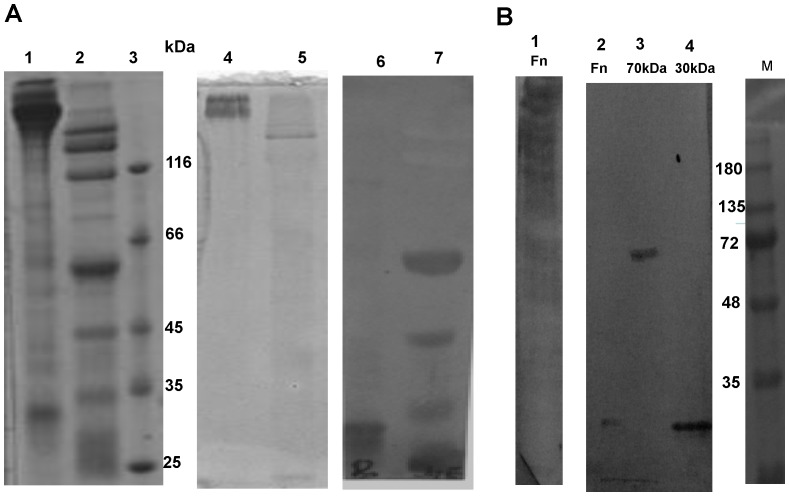
Fn52 binds to cryptic regions in the fibronectin. Panel A: Reducing SDS-PAGE, stained with Coomassie Blue. Lane 1: fibronectin (Sigma Chemicals), Lane 2 : the same fibronectin, post-cleavage with collagenase, Lane 3 shows the indicated molecular weight markers. Lanes 4 and 5 represent the corresponding gel transferred to nitrocellulose membrane, and stained with amido black. Lanes 6 and 7 represent western blot images of lanes 1 and 2 respectively, using scFv Fn52 as primary antibody and mouse anti-His monoclonal antibody, followed by anti-mouse HRP conjugated secondary antibody. Panel B: Western blot images of full length fibronectin (lane 1), using anti-fibronectin antibody (Sigma Chemicals); lanes 2, 3 and 4 show the signals obtained using scFv Fn52 as primary antibody for full length fibronectin (lane 2), 70 kDa fragment of fibronectin (lane 3) and the 30 kDa fragment of fibronectin (lane 4) respectively. M represents the molecular weight marker lane.

In order to determine whether the scFv binds to native fibronectin, or a region which is cryptic within the molecule, we cleaved fibronectin with recombinant collagenase derived from *Vibrio mimicus*. Vibrio metalloproteases are known to share similar substrate specificities [22]; the metalloprotease derived from *Vibrio cholera* has been shown to hydrolyze fibronectin [23]. Our results show that collagenase derived from *V. mimicus*, can also similarly hydrolyze fibronectin (Figure 3, Panel A, lane 2). When probed with the scFv Fn52 in a western blot, following SDS-PAGE under reduced conditions, binding of the scFv is seen to occur only with the lower molecular weight fragments of fibronectin, both in the case of whole fibronectin molecule (Figure 3A, lane 6), as well as collagenase-treated fibronectin (lane 7). Fn52 does not show any binding to the intact molecule of fibronectin (Figure 3A, lane 6), although the transfer of the protein to the membrane was confirmed by amido black staining of the membrane (Figure 3A, lanes 4 and 5). This is to be expected, since the scFv was generated through screening against the lower molecular weight fragment (30-kDa) of fibronectin. Even so, binding of the scFv was more prominent in the case of collagenase-treated fibronectin.

A western blot of the 70 kDa and 30 kDa fragments of fibronectin sourced commercially (Figure 3, Panel B, lanes 3 and 4 respectively), using the scFv Fn52, also showed binding of the scFv to the 70 kDa and the 30 kDa fragments independently. No binding of Fn52 was observed with the intact molecule of fibronectin (Figure 3B, lane 2); however, anti-fibronectin antibody (Sigma Chemicals), was observed to bind to the intact fibronectin (Figure 3B, lane 1). The above results suggest therefore, that the scFv recognizes some cryptic residues within the fibronectin molecule, exposure of which occurs upon cleavage of fibronectin to generate smaller fragments of the molecule. It may be noted that the irrelevant control scFv (O27) used in the study, did not result in any binding to either the intact fibronectin or its fragments (Figure S4 in File S1).

The binding of the scFvFn52 to RPE cells was also confirmed by immunocytochemistry using Fn52 as the primary antibody, followed by FITC-labeled mouse anti-c-myc antibody as the secondary antibody. Once again, O27 did not show any binding to the RPE cells (Figure S5 in File S1).

### Fn52 and Fn52RGDS do not Result in any Change in Cell Viability, But Cause Reduction in Cell Proliferation

**Figure 4:**
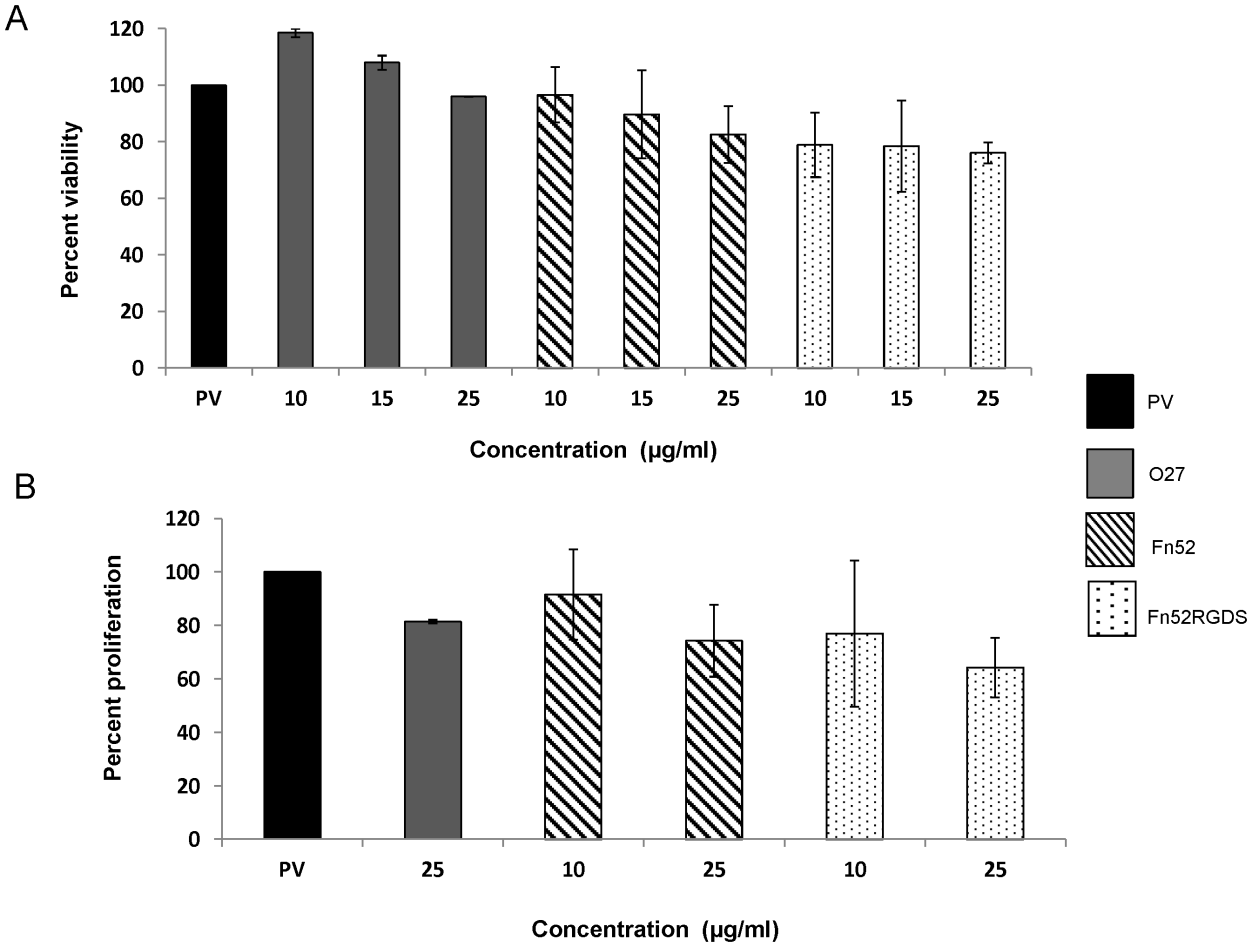
Changes in cell viability and proliferation in the presence of Fn52 and Fn52RGDS. Panel A: D407 RPE cell viability assessed by the MTT assay. The optical density of the wells containing cells seeded in the presence of patient vitreous/SRF was assigned as 100%. Panel B: RPE cell proliferation was assessed by the BrdU assay. PV (assigned as 100%) represents conditioned media obtained from cells grown in the presence of patient vitreous (and subretinal fluid). In both the assays, the control scFv O27 was also used; the effect of the scFv antibodies was evaluated, using a range of concentrations. Each bar represents mean±SEM (standard error of the mean) calculated from five and three separate experiments respectively.

MTT assay was used to assess the viability of RPE cells in the presence of the two antibodies. D407 RPE cells were cultured in the presence of vitreous and subretinal fluid samples (labeled as “PV”) obtained from patients with phakic/pseudophakic rhegmatogenous retinal detachments undergoing retinal re-attachment surgery. This is because we have observed that such samples have a potential to cause fibrotic responses (unpublished results). The results are shown in Figure 4A; it can be seen that PV supports viability of the D407 RPE cells, although no FBS has been added to these cultures. Further, although the viability of cells was reduced in the presence of increasing concentrations of both the scFv antibodies (over the range of 0–50 µg/ml), this decrease was not statistically significant. Since it was observed that the cells did not survive at antibody concentrations of 50 µg/ml, over a longer time scale (48 h) (data not shown), we decided to study the effect of these antibodies at a concentration of 25 µg/ml for the various assays.

BrdU incorporation was used to assess the extent of proliferation of the RPE cells. It was observed that in the absence of any scFv antibody, the cells were able to proliferate in the presence of vitreous/subretinal fluid sampled from the patients, indicating that the vitreous contains sufficient growth factors to support this proliferation (as reported earlier [24]), over and above the proliferation seen in the presence of FBS (data not shown). At a concentration of 25 µg/ml, both the scFv antibodies were able to reduce the proliferation of RPE cells in the presence of vitreous/SRF samples (Figure 4B). Proliferation in the presence of patient-derived vitreous and subretinal fluid was taken to be 100%; scFv Fn52 reduced the proliferation to 74%; scFv Fn52RGDS reduced the proliferation to 64%; the decrease was however not found to be significant.

The irrelevant control antibody, scFv O27 had almost no effect on either the viability or the proliferation of the D407 RPE cells.

### The scFv Antibodies do not Support RPE Cell Adhesion

**Figure 5:**
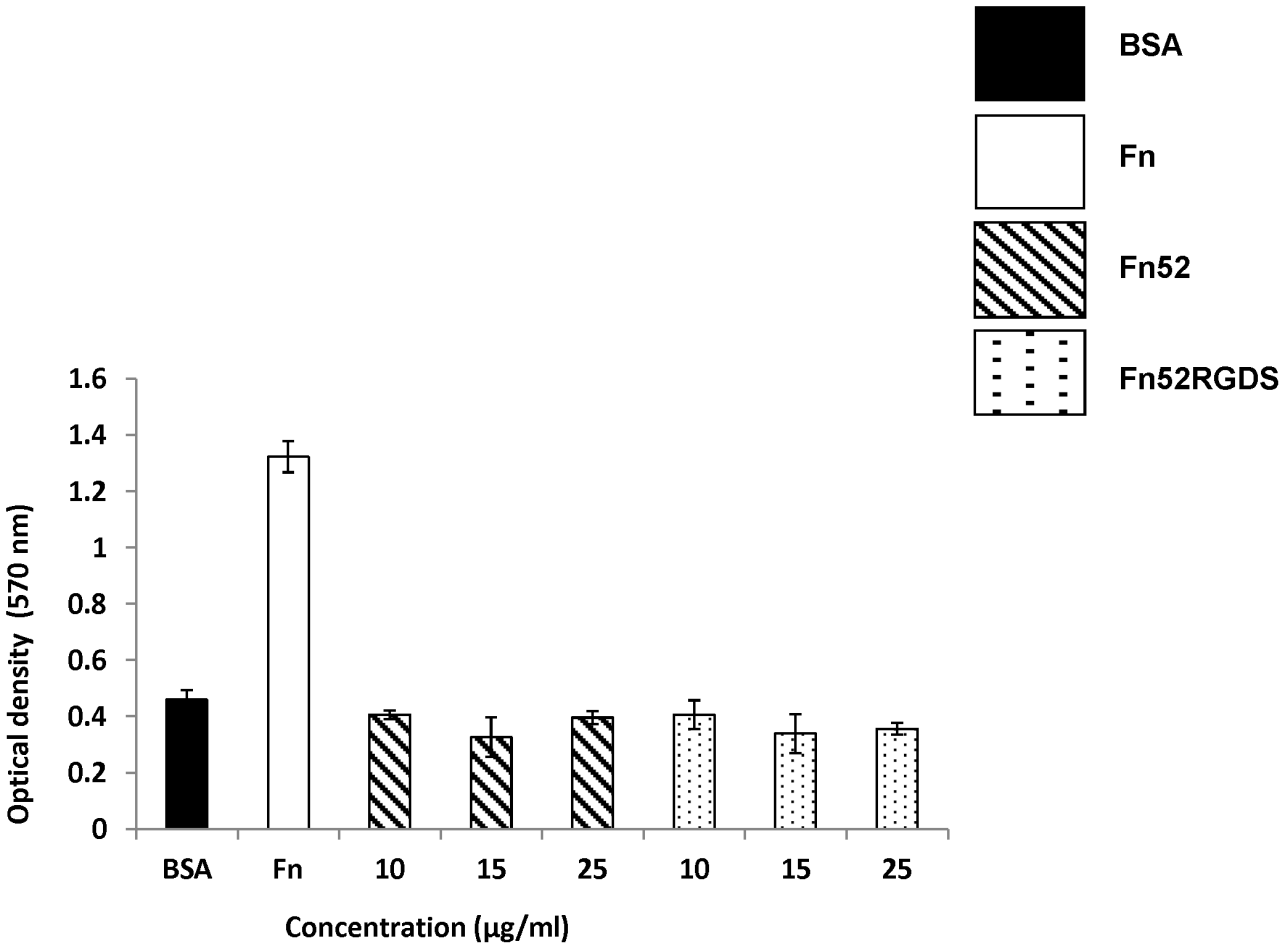
The scFv antibodies do not support RPE cell adhesion. Adhesion assay performed to assess the adhesion of D407 RPE cells over wells that were left uncoated (blocked with BSA only-negative control), or coated with fibronectin (Fn, at a concentration of 25 µg/ml; positive control), or with a range of concentration of the two scFv antibodies. Each bar represents mean±SEM (standard error of the mean) calculated from three separate experiments.

Figure 5 shows that when the wells were coated with the two antibodies, Fn52 and Fn52RGDS over a range of concentrations (0–25 µg/ml), the numbers of D407 RPE cells attached for Fn52 and Fn52RGDS were similar to that in BSA-coated wells (negative control), showing that these scFv antibodies do not support adhesion, unlike the adhesive protein, fibronectin (used as a positive, adhesive control).

### Both the scFv Antibodies Result in Reduced Cell Migration

**Figure 6:**
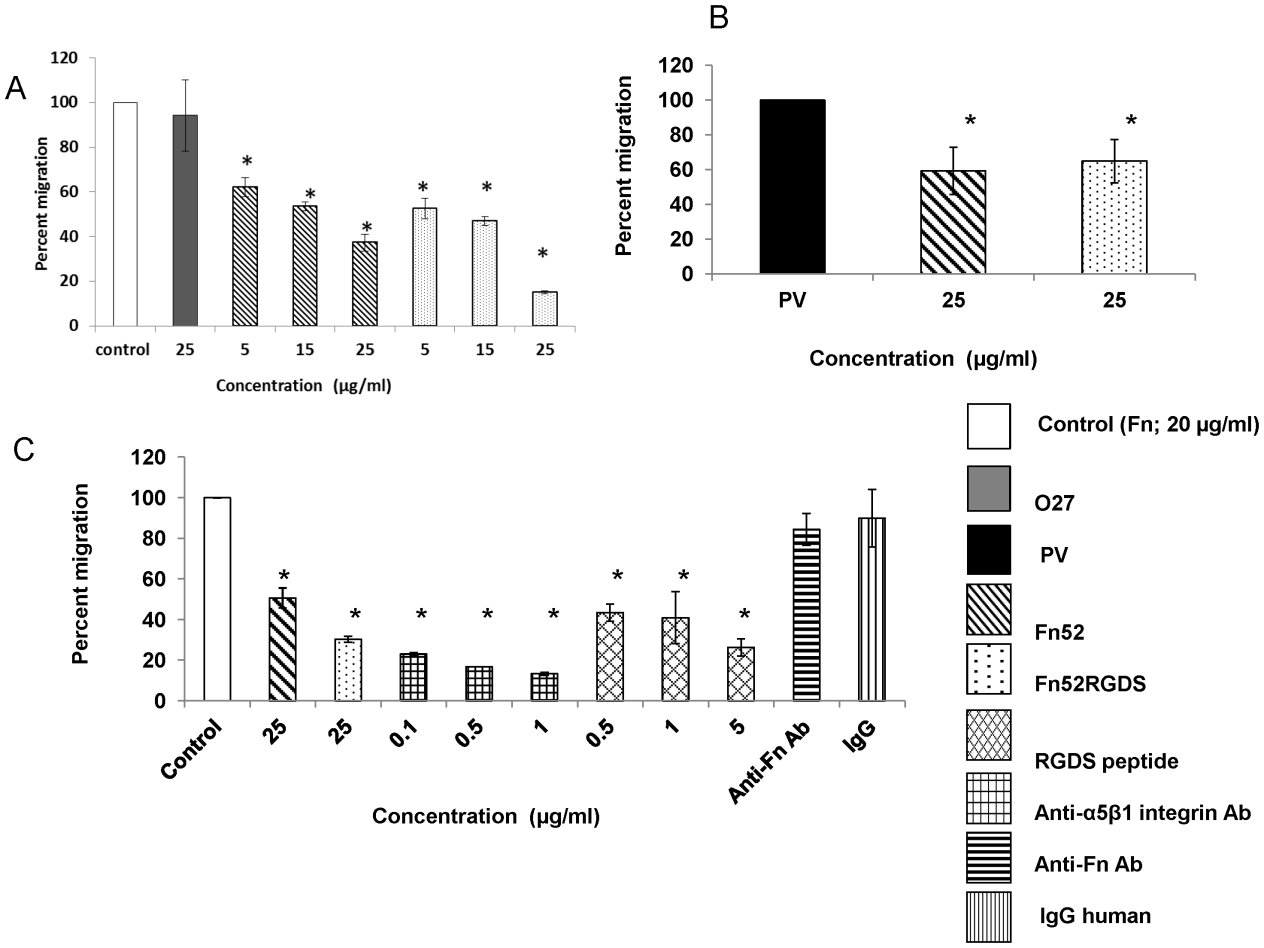
The effect of Fn52 and Fn52RGDS on migration of D407 RPE cells. Panel A: The migration of D407 RPE cells was assessed in response to fibronectin, in the presence of Fn52, Fn52RGDS as well as O27 (negative control).Panel B: Migration of D407 RPE cells in response to patient-derived vitreous and subretinal fluid (PV) was evaluated in the presence of 25 µg/ml of Fn52 and Fn52RGDS respectively. Panel C: RPE cells were pre-incubated for 1 h with the various reagents, at indicated concentrations, and added to the upper chamber of the transwell insert, while the bottom chamber in each well, contained Fn at a concentration of 20 µg/ml (control). In each case, the results are expressed as mean± standard deviation; P<0.05 (one-way ANOVA).

Figure 6 shows the migration of RPE cells in transwell inserts under the influence of fibronectin(Panel A) or patient vitreous/SRF (Panel B) used as a chemotactic factor. The scFv antibodies were tested for their ability to inhibit this migration. As is evident from Figure 6A, at a concentration of 25 µg/ml, the scFv Fn52RGDS was able to effect a more than 2 fold decrease in migration as compared to scFv Fn52 (Fn52: 37%; Fn52RGDS:15%). The migration of RPE cells in the presence of the control scFv, O27, was comparable to that seen in the presence of fibronectin alone. In the presence of patient-derived vitreous (and subretinal fluid), the two scFv antibodies (at 25 µg/ml), were similarly and significantly effective in reducing the migration of RPE cells(Fn52: 66%; Fn52RGDS:73%) (Figure 6B).

### scFv Fn52RGDS Binds to Integrin

Since both, RGD peptides, as well as anti-integrin antibodies can bind to integrins, and block fibronectin-intergin interactions, we carried out migration experiments using these reagents in a dose-dependent fashion, to compare the relative migration of RPE cells in the presence of the two scFv antibodies, differing only by an additional RGD sequence stretch (Figure 6C). For this purpose, RPE cells were pre-incubated with different reagents as described in the methods section. These cells were seeded in the upper chamber of the transwell inserts and the lower chamber contained fibronectin, as the chemoattractant. As seen from figure 6C, when the RPE cells were pre-incubated with the anti-integrin antibodies, there was decreased migration of cells, as compared to the case when cells were pre-incubated with RGD peptides although, in both cases, the reduction was statistically significant in the entire range of doses tested (0.1–1.0 µg/ml for anti-integrin antibody and 0.5–5.0 µg/ml for the peptide). Clearly, blocking of the cell surface integrins by any of the above two methods, results in reduced migration of the cells. Similarly, when the RPE cells were pre-incubated with either Fn52 or Fn52RGDS, there was greater reduction in migration in the case of Fn52RGDS (30.3%), as compared to the decrease seen in presence of Fn52 (50.6%). It may therefore be inferred that the reduction in migration effected by the scFv Fn52RGDS in comparison with that of scFv Fn52, must be due to the presence of the “RGD” sequence which could be blocking the integrin-fibronectin interactions. Two control antibodies were included in the experiment: the human IgG antibody, which did not result in any reduction in migration; interestingly, the antibody against fibronectin (Sigma Chemicals), which binds to fibronectin (Figure S6 in File S1), also did not result in any change in migration of the RPE cells.

### Assessment of Fibronectin Polymerization in the Presence of the scFv Antibodies

**Figure 7:**
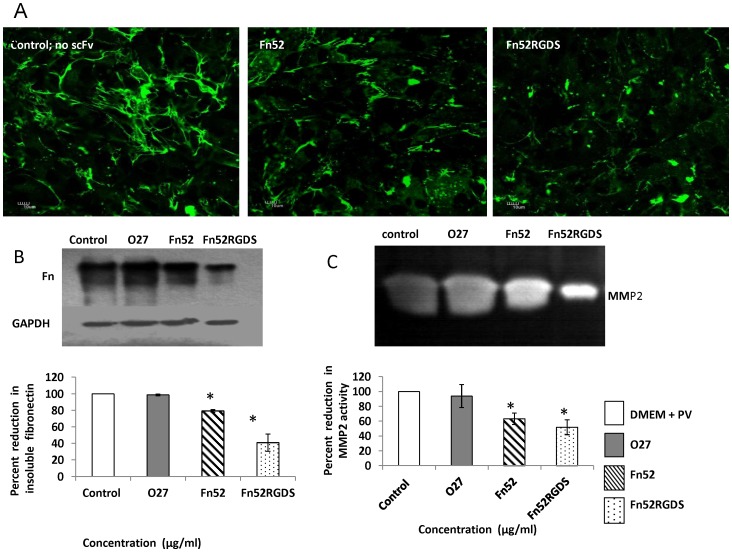
Effect of Fn52 and Fn52RGDS on fibronectin polymerization and MMP expression in ARPE-19 RPE cells. Panel A: Immunocytochemistry of ARPE-19 retinal pigment epithelial cells, cultured in the presence of patient-derived vitreous and subretinal fluid, in the presence and absence of scFv antibodies. Staining was done using rabbit anti-fibronectin antibody (Sigma; F3648);FITC-labeled anti-rabbit antibody was used as secondary antibody. Images (60×) were acquired on an Olympus confocal laser scanning microscope. Scale bars represent 10 µm. Panel B: Insoluble fibronectin content in ARPE-19 retinal pigment epithelial cells, cultured as described in Panel A above, was analyzed by deoxycholic acid (DOC) used for the solubilization of Fn multimers, which were generated starting with the same number of cells in each case (control and scFv-treated wells).The blots were developed using anti-fibronectin antibody (1∶5000;Sigma) by chemiluminescence (Amersham Biosciences). The DOC-soluble fraction was analyzed by immunoblotting using mouse anti-GAPDH antibody, as internal control. The figure is a representative image of three separate experiments. Densitometric quantitation of immunoblotting was done, and analysed by the Image-J software; the figure represents mean±SEM (standard error of the mean) calculated from three separate experiments. Lane 1: control - cells in the presence of DMEM and patient-derived vitreous and subretinal fluid (PV); lanes 2–4: cells grown in the presence of PV and 25 µg/ml of O27, Fn52 and Fn52RGDS respectively. Panel C: Zymogram of culture medium supernatants obtained upon culturing ARPE-19 (retinal pigment epithelial cells) cells as described in Panel A, in the absence or presence of scFv antibodies (lane 1: no scFv; lane 2: scFvO27; lane 3: scFv Fn52; lane 4: scFv Fn52RGDS). Densitometric quantitation of the signals in the zymogramwas done, and analysed by the Image-J software; the figure represents mean±SEM (standard error of the mean) calculated from four separate experiments.

RPE cells are known to synthesize fibronectin at low levels [25], [26]; however, the displacement of the RPE cells from the normal location, causes increased synthesis of fibronectin, as in the case of proliferative vitreoretinopathy. In order to test the changes in fibronectin expression, we chose to use the ARPE-19 retinal pigment epithelial cells, since we found that this RPE cell line (unlike the D407 RPE cells) is capable of appreciable synthesis of fibronectin in the presence of patient-derived vitreous and subretinal fluid. Staining of ARPE-19 cells in the presence of the different constructs is shown in Figure 7A. It is clear from these images that i) ARPE-19 RPE cells synthesize fibronectin in the presence of PV; both cellular fibronectin and intercellular fibrils were visible; ii) addition of scFv Fn52 results in reduction of fibronectin polymerization; iii) addition of scFv Fn52RGDS results in protofibrils not progressing to the fibrillar form, and the cell-associated fibronectin is also less formed. The negative control (absence of any added scFv antibody), as well as staining images using the unrelated antibody, O27, is presented in Figure S7 in File S1. Since it is difficult to appreciate the changes in fibronectin polymerization by the immunostaining data, we carried out the DOC assay (Figure 7B) to quantitate the changes in levels of insoluble fibronectin. Figure 7B shows that Fn52RGDS was more effective in lowering the level of fibronectin polymerization (41%), as compared to Fn52 (79.3%). The irrelevant control scFv O27, did not result in any significant change in fibronectin polymerization (98.6%). In each case, anti-GAPDH antibody was used to probe the soluble fraction, as internal control, to ensure equal protein loading of all the wells.

### Examination of Levels of MMP

Since changes in levels of fibronectin polymerization were clearly seen in the case of ARPE-19 cells, we investigated the levels of MMPs generated by these cells (Figure 7C) in the absence (lane 1) or the presence of the two scFv antibodies (lanes 3 and 4 respectively). Figure 7C shows that expression of MMP2 is significantly reduced upon exposure to the scFv antibodies; the irrelevant scFv O27, did not lead to any change in level of MMP2 expression (lane 2; 93.8%).Expression of MMP2 was significantly reduced in the presence of both scFv Fn52 (63.2%) and Fn52RGDS (51.9%).

### Collagen Gel Contraction

**Figure 8:**
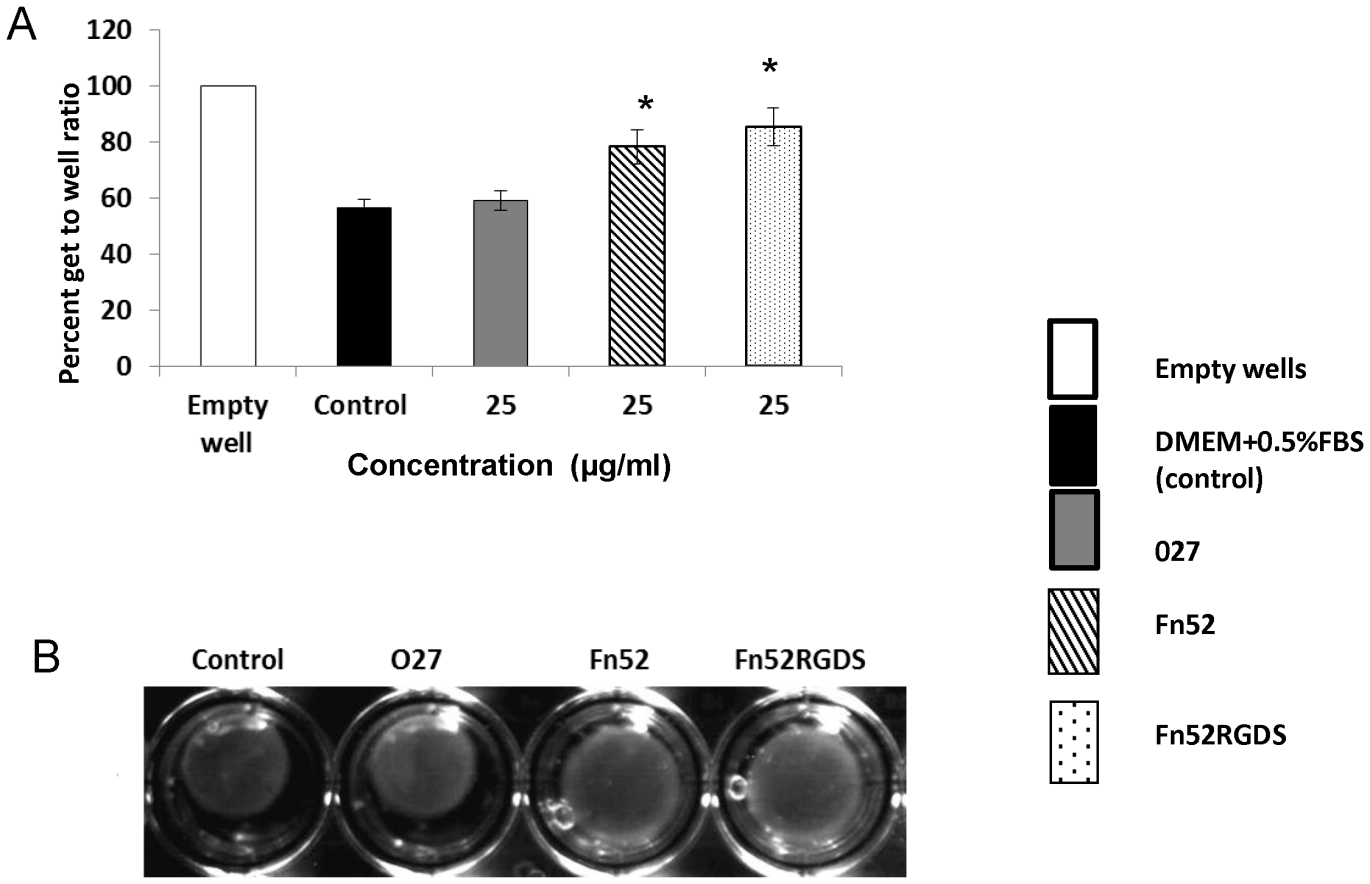
Analysis of collagen gel contraction in the presence of the scFv antibodies. Collagen gel contraction was performed using HT-1080 cells, to evaluate contraction of gel in the absence of any added scFv, or in the presence of either O27 or Fn52 or Fn52RGDS. Pictures were recorded 4 h after overlaying with media (representative image of four separate experiments) (Panel A). The area of the collagen gels was measured and expressed as gel/well ratio (%). The data is represented as mean±SEM (standard error of the mean) calculated from four separate experiments (Panel B).

In the context of PVR, the RPE cells proliferate and migrate over the vitreal surface of the retina, thereby forming a contractile connective tissue membrane. This membrane generates a contractile force, which results in detachment of the retina. Fibroblast-mediated collagen gel contraction assays have been used as an *in vitro* model of tissue contraction which characterizes fibrosis. HT-1080, a human fibrosarcoma cell line, is derived from fibrous connective tissue, characterized by the presence of proliferating fibroblasts, and has been used in such assays [27]. Collagen gel contraction using HT-1080 cells showed that, both Fn52 and Fn52RGDS are equally effective in inhibiting this contraction (Figure 8); after 4 h of overlay of media, the control well (in absence of any added scFv) resulted in a contraction of 43.3%, while the levels of contraction in the presence of Fn52 and Fn52RGDS were 21.75% and 14.5% respectively. O27 on the other hand showed a contraction (41%), comparable to the control.

## Discussion

Currently, the management of PVR mainly involves surgical intervention, but the patients often suffer from considerable visual loss. In addition therefore, several pharmacologic agents are being explored to prevent PVR. These include use of systemic corticosteroids, anti-neoplastic drugs, polyphenolic compounds, inhibitors of cytokines and growth factors and others [28]–[33].

In this paper, we have designed an antibody targeted specifically at fibronectin, since it is an important component of the fibrous matrix surrounding the RPE cells of the epiretinal membranes (ERM) [34], [35], the contraction of which leads to tractional retinal detachment (TRD) [36].It has been seen that fibronectin is the major component in the ERM in both fibrillar and pericellular arrangement; increased levels of fibronectin were observed in both subretinal fluid and pathologic vitreous; and intravitreal fibronectin concentration was observed to increase with clinical stages of evolution of PVR [37]. This suggested that fibronectin could have an important role in earliest pre-membranogenic stages of PVR condition, and also that it could modulate the adhesion mechanisms in established membranes, thus making fibronectinin dispensable in PVR. Indeed Ikuno and Kazlauskas [38] have also suggested that the excessive amount of extracellular matrix in the epiretinal membrane may be considered to be a particularly important component of the progression of PVR. Since alterations in the ECM are known to modulate cellular behavior such as proliferation, migration, differentiation and adhesion of cells in the context of PVR [34], we attempted to target the fibronectin component of the ECM.

Some attempts have been made earlier to block Fn matrix assembly without affecting the initial binding of Fn to cell surface receptors (such as α5β1) (Figure 1). This includes the use of either i) monoclonal antibodies (such as L8 or 9D2), directed against Fn domains embedded within, or near, the Fn III 1,2 modules that are critical for Fn polymerization, or ii) competitors of Fn polymerization (such as the N-terminal 70 kDa region of Fn or the 49-mer peptide derived from the Streptococcus pyogenes adhesion F1 protein), which compete for matrix assembly sites on cell surfaces [11], [39]–[41].Yet other agents that can block fibronectin matrix assembly are those that play a role in the disruption of the binding of soluble fibronectin to α5β1. These include the use of i) anti-integrin antibodies or ii) RGD peptides which again act as competitive inhibitors [42]–[44]. However, none of these have been used earlier in the context of PVR.

It is conceivable to have Fn polymerization blocking agents capable of blocking more than one potential assembly site, or alternatively having more than one blocking agent, capable of simultaneously blocking distinct putative assembly sites in Fn. Our study incorporates the use of the former approach, i.e., combining the above two pathways of blocking Fn assembly i.e., blocking both, the self-assembly sites, as well as, the disruption of Fn-cell surface binding, and to the best of our knowledge, it is the first study which exploits the use of a single antibody capable of targeting two important Fn polymerization sites: the N terminal 30-kDa region and the RGD site. There are currently no known antibodies against the N-terminal 30 kDa region of fibronectin, which are capable of blocking Fn polymerization.

The RPE cells *in situ*, are known to synthesize very low levels of fibronectin [16]. The upregulation of fibronectin is important in the context of PVR, when the RPE cells have been displaced from their normal location, and are exposed to components of the serum. In order to assess the changes in fibronectin matrix assembly, we examined the level of fibronectin synthesis using two different RPE cell lines – D407 and ARPE-19. Both show minimal levels of fibronectin expression; however, it was found that ARPE-19 cells synthesize appreciable amounts of fibronectin upon stimulation with patient-derived vitreous (Figure 7). Similar results have been seen earlier with this cell line when the cells were stimulated with TGF-β [26]. Therefore, for the fibronectin polymerization and MMP2 expression studies, we chose to work with the ARPE-19 RPE cells. In order to examine the changes in collagen gel contraction, HT-1080 cell line (a fibrosarcoma cell line, characterized by the presence of proliferating fibroblasts),was chosen, because such cultures have been used to model tissue contraction [27],and can best characterize the events that take place in wound repair and fibrosis; also, PVR involves proliferation of various cell types, including RPE, macrophages, astrocytes and fibroblasts, on the inner surface of the retina and within the vitreous.

It is well established that fibronectin matrix assembly i) enhances adhesion-dependent cell growth [7], ii) facilitates integrin-mediated cell contractility [8], and iii) controls the stability and composition of ECM and of cell-matrix adhesion sites [45], and thus may be responsible for regulating many aspects of cell behavior.Our results indicate that the scFv antibody, Fn52RGDS, which causes greater inhibition of fibronectin matrix assembly (compared to Fn52), also causes decreasedcell migration and decreased MMP activity, compared to Fn52. scFv Fn52RGDS is thus better than the parent antibody, Fn52, possibly because of the additional RGD sequence stretch, which allows the scFv to block fibronectin-integrin interactions (Figure 6C), besides inhibiting Fn matrix assembly. The effect on matrix metalloproteases is in line with previous studies showing that fibronectin induces MMP-9 expression in HEp-2 cells, mediated through the integrin receptor alpha5beta1, and involves activation of multiple signaling pathways [46]. It has also been reported that fibronectin induces MMP-2 and MMP-9 production in T-cell lines, mediated by engagement of fibronectin receptors (such as α4,α5 and αv) [47]. Inhibition of fibronectin assembly would thus be expected to cause decreased expression of MMP levels, as is the case with the two antibodies; inhibition of integrin-fibronectin interactions would be expected to cause a further decrease in MMP levels, which is seen in the case of the scFv, Fn52RGDS.

**Figure 9:**
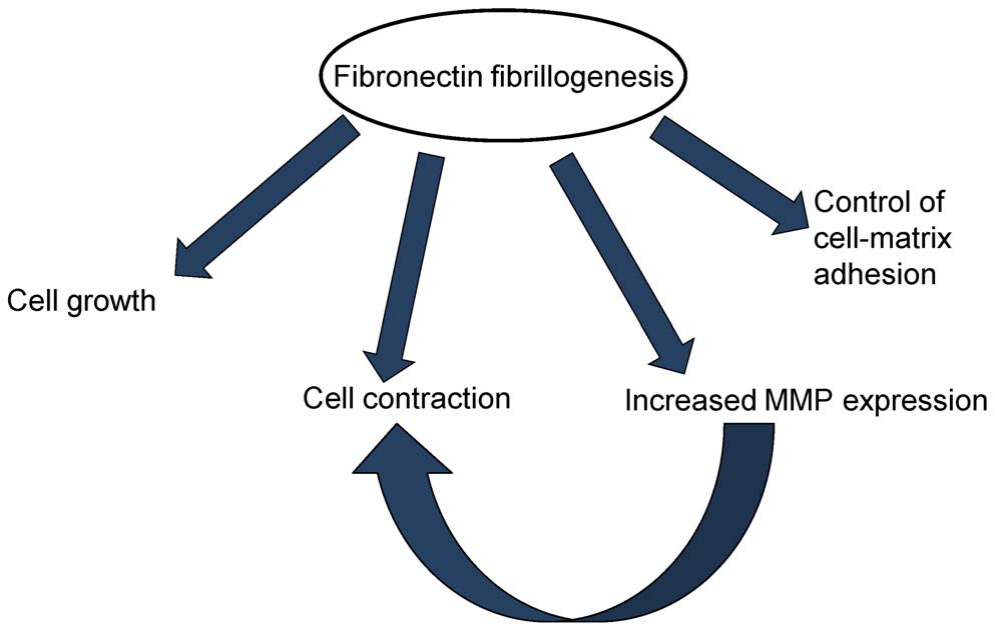
The importance of fibronectin matrix assembly in various cellular processes. Fibronectin matrix assembly regulates cell growth, cell matrix adhesion, cellular contraction, and matrix metalloproteases (MMP) expression. Decreased fibrillogenesis resulting in decreased MMP expression may itself also be responsible for inhibition of cellular contraction. The scFv Fn52RGDS acts in accordance with features suggestive of decreased fibrillogenesis.

The test of the inhibitory properties of the scFv antibodies is best exemplified by the collagen gel contraction assay, which is the *in vitro* simulator of vitreal contraction in vivo [34]. This contraction occurs through the attachment of fibroblasts to the collagen via the integrins, followed by degradation of the collagen by MMPs. Thus MMPs play an important role in three-dimensional matrix contraction, as in PVR membrane contraction. Indeed MMP inhibition has been shown to reduce matrix contraction involving fibroblasts [48] and retinal pigment epithelial cells [49]. Further, it has also been observed that cellular fibronectin is required by the fibroblasts for collagen gel contraction [50]. Our data is in line with these results; decreased expression of fibronectin by the two antibodies could be resulting in decreased collagen gel contraction; alternatively, decreased MMP expression (due to decreased expression of fibronectin), could also be responsible for inhibition of collagen gel contraction (schematic shown in Figure 9).

In conclusion, the desirable properties of the scFvFn52RGDS appear to result from the fact that i) the parent scFv (Fn52) binds to a cryptic epitope within the fibronectin molecule, exposure of which may be important during fibronectin matrix assembly, and also in the course of some of the cellular processes such as cell adhesion, migration, proliferation and differentiation; ii) the sequence tag “RGDS” inhibits fibronectin-integrin interactions, and this appears to be crucial in mediating the characteristic features that define a fibrotic pathology, since this antibody is different from the parent antibody (Fn52) in only this additional stretch of amino acids. Our data provides evidence of the therapeutic relevance of such scFv antibodies in controlling fibroproliferative diseases. This is particularly attractive given the fact that these antibodies a) have lack of cytotoxicity, b) are low molecular weight antibodies, c) are of fully human origin, andd) have the potential to inhibit all the features of the pathology (adhesion, migration, contraction, MMP secretion). The use of Fn52RGDS as a “double-edged sword” (targeting both, the fibronectin molecule, as well as the interaction of fibronectin with the cell surface receptors, ie., integrins through the RGD sequence), can be effective not only in the context of fibrosis as in PVR, but also in other pathological situations such as tumors and thrombosis. Experiments with animal models of PVR need to be evaluated next, to further prove their potential.

## Supporting Information

File S1 **Table S1.** **Enrichment of phages from Library J showing binding to N terminal 30 kDa Fn fragment during four subsequent biopanning steps. Figure S1.** **Schematic diagram illustrating the cloning strategy for re-engineering of Fn52 phagemid clone to the RGDS-containing Fn52RGDS clone.** **Figure S2.** **Cell ELISA assay data with different scFv antibody clones.** The figure represents binding of D407 RPE cells to 17 different scFv antibody clones picked up at random. Two clones in the histogram (red and blue bars) showed maximal binding to RPE cells. One of these, designated scFv Fn52 (corresponding to the red bar) showed reproducible binding to target cells. **Figure S3.** **SDS-PAGE profiles.** Purified scFv Fn52 (lane 2; Panel A); the corresponding western blot using anti-His antibody (lane3; Panel A). Panel B represents the SDS-PAGE profile of purified fraction of scFv Fn52RGDS. Lane 1 in both panels shows the indicated molecular weight markers. **Figure S4.** **Western blot images using scFvO27 as primary antibody.** Full length fibronectin (lane 1), 70 kDa fragment of fibronectin (lane 2) and the 30 kDa fragment of fibronectin (lane 3) respectively. M represents the molecular weight marker lane. **Figure S5.** **Binding of scFv Fn52 and O27 to D407 RPE cells**. Immunocytochemical staining, using the scFv antibodies as primary antibody, followed by mouse anti-c-myc FITC-labeled antibody as secondary antibody. Negative control (minus primary antibody) is also shown. The bottom panels are the corresponding images with the nuclear marker, Hoechst dye. The magnification bar corresponds to 50 µm. **Figure S6.** **Fibronectin in D407 RPE cells.** Immunocytochemistry of D407 RPE cells carried out to stain fibronectin in presence of DMEM plus 10%FBS, using rabbit anti-fibronectin antibody (Sigma; F3648), followed by anti-rabbit FITC-labeled antibody as secondary antibody. Negative control (minus primary antibody) is also shown. The bottom panels are the corresponding images with the nuclear marker, Hoechst dye. The magnification bar corresponds to 50 µm. **Figure S7. Fibronectin in ARPE-19 RPE cells.** Immunocytochemistry of ARPE-19 RPE cells stained with rabbit anti-fibronectin antibody (Sigma; F3648), in the absence or presence of three scFv antibodies, O27, Fn52 and Fn52RGDS. FITC-labeled anti-rabbit antibody was used as secondary antibody. DAPI staining was done for nuclear staining. An image with no primary antibody (negative control) has been included. Images were acquired (60×) on an Olympus confocal laser scanning microscope system.
(PDF)
