# Supplementary material for: Fibrotic Remodeling of the Extracellular Matrix through a Novel (Engineered, Dual-Function) Antibody Reactive to a Cryptic Epitope on the N-Terminal 30 kDa Fragment of Fibronectin
Source: PLoS One. 2013 Jul 23;8(7):e69343. doi: 10.1371/journal.pone.0069343 (PMC3720593; doi:10.1371/journal.pone.0069343)
Supplement: File S1 — Table S1. Enrichment of phages from Library J showing binding to N terminal 30 kDa Fn fragment during four subsequent biopanning steps. Figure S1. Schematic diagram illustrating the cloning strategy for re-engineering of Fn52 phagemid clone to the RGDS-containing Fn52RGDS clone. Figure S2. Cell ELISA assay data with different scFv antibody clones. The figure represents binding of D407 RPE cells to 17 different scFv antibody clones picked up at random. Two clones in the histogram (red and blue bars) showed maximal binding to RPE cells. One of these, designated scFv Fn52 (corresponding to the red bar) showed reproducible binding to target cells. Figure S3. SDS-PAGE profiles. Purified scFv Fn52 (lane 2; Panel A); the corresponding western blot using anti-His antibody (lane3; Panel A). Panel B represents the SDS-PAGE profile of purified fraction of scFv Fn52RGDS. Lane 1 in both panels shows the indicated molecular weight markers. Figure S4. Western blot images using scFvO27 as primary antibody. Full length fibronectin (lane 1), 70 kDa fragment of fibronectin (lane 2) and the 30 kDa fragment of fibronectin (lane 3) respectively. M represents the molecular weight marker lane. Figure S5. Binding of scFv Fn52 and O27 to D407 RPE cells. Immunocytochemical staining, using the scFv antibodies as primary antibody, followed by mouse anti-c-myc FITC-labeled antibody as secondary antibody. Negative control (minus primary antibody) is also shown. The bottom panels are the corresponding images with the nuclear marker, Hoechst dye. The magnification bar corresponds to 50 µm. Figure S6. Fibronectin in D407 RPE cells. Immunocytochemistry of D407 RPE cells carried out to stain fibronectin in presence of DMEM plus 10%FBS, using rabbit anti-fibronectin antibody (Sigma; F3648), followed by anti-rabbit FITC-labeled antibody as secondary antibody. Negative control (minus primary antibody) is also shown. The bottom panels are the corresponding images with the nuclear marker, Hoechst dye. The [file pone.0069343.s001.pdf]

Table S1

Round of biopanning	Input phages	Eluted phages (Tu/ml)
First	$7X10^{11}$	$1.7X10^{5}$
Second	$1X10^{11}$	$2.6X10^{6}$
Third	$1X10^{11}$	$1.2X10^{7}$
Fourth	$1X10^{11}$	$1.2X10^{6}$

Figure S1

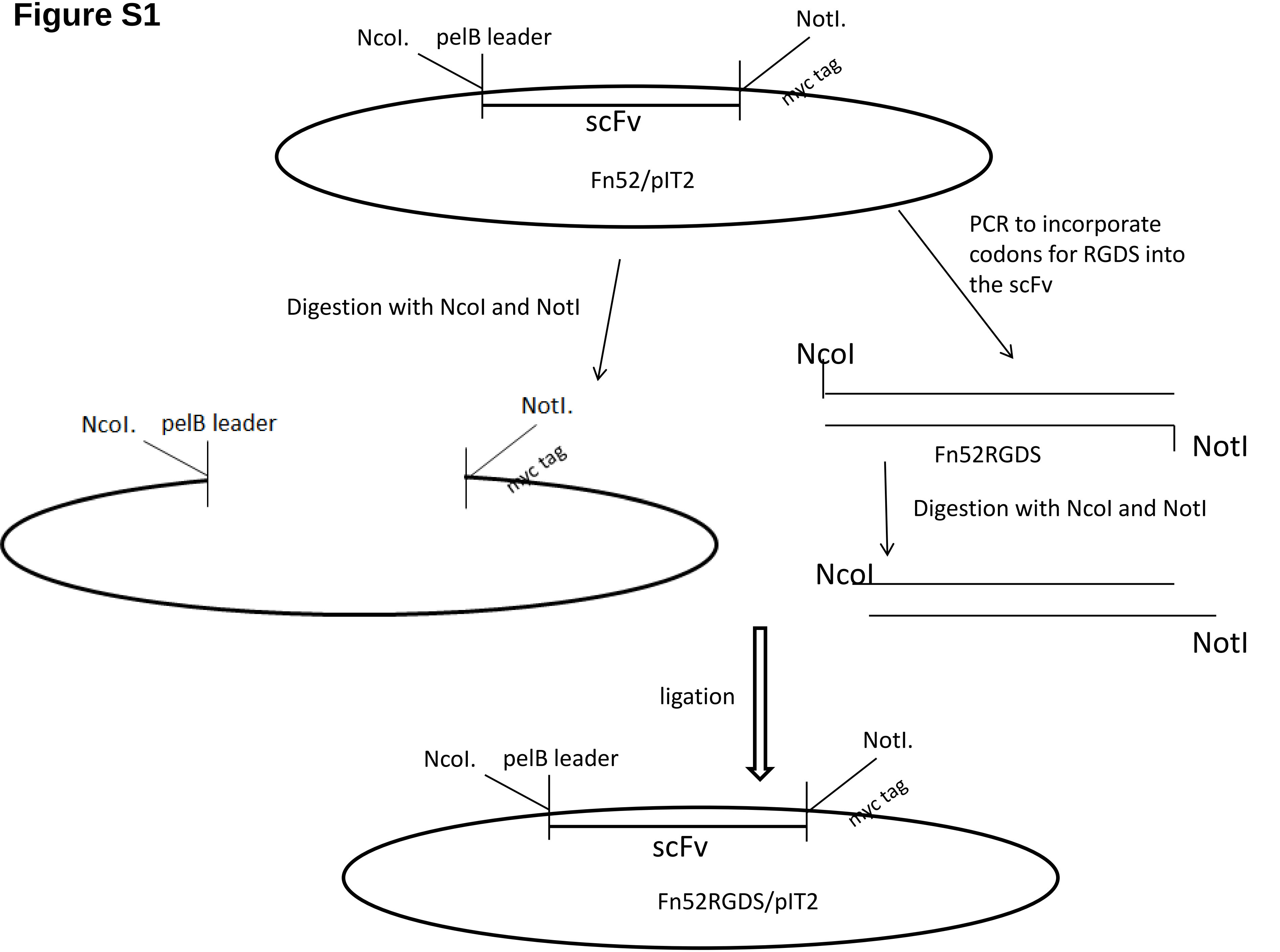
NcoI.
pelB leader
NotI.
myc tag
scFv
Fn52/pIT2
PCR to incorporate codons for RGDS into the scFv
Digestion with NcoI and NotI
NcoI
NotI.
NcoI.
pelB leader
myc tag
Fn52RGDS
NotI
Digestion with NcoI and NotI
NcoI
NotI
ligation
NotI.
NcoI.
pelB leader
myc tag
scFv
Fn52RGDS/pIT2

Figure S2

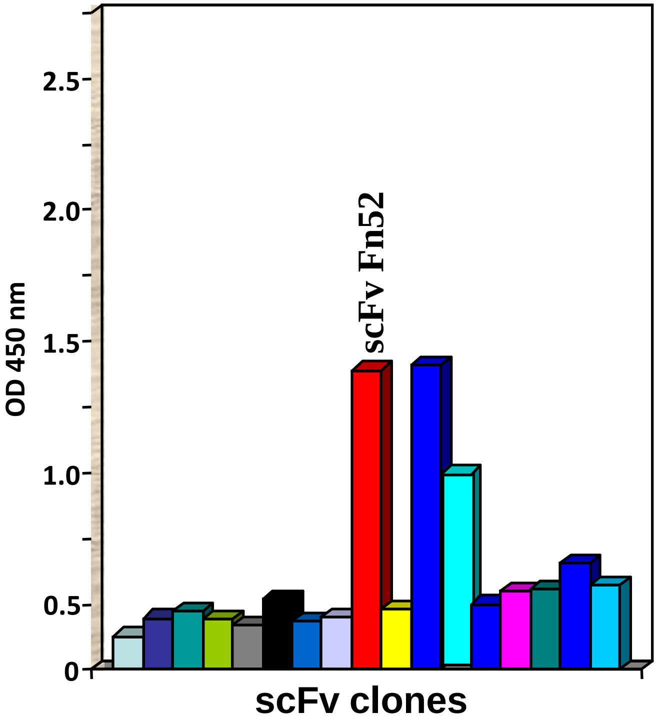

Figure S3

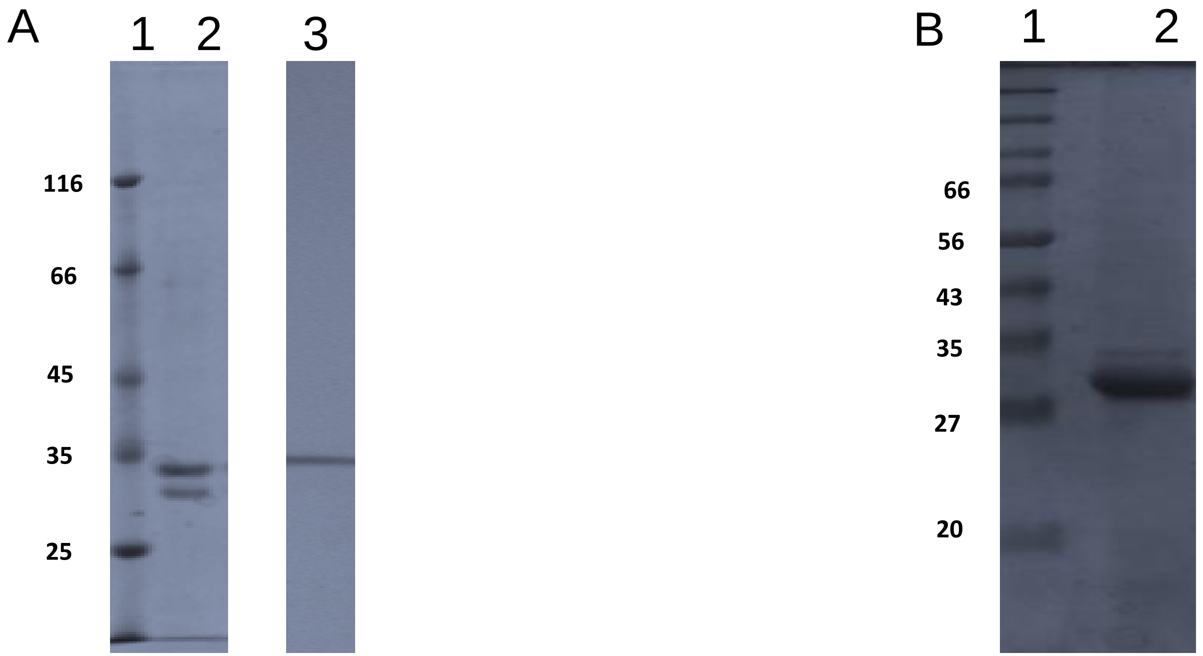

Figure S4

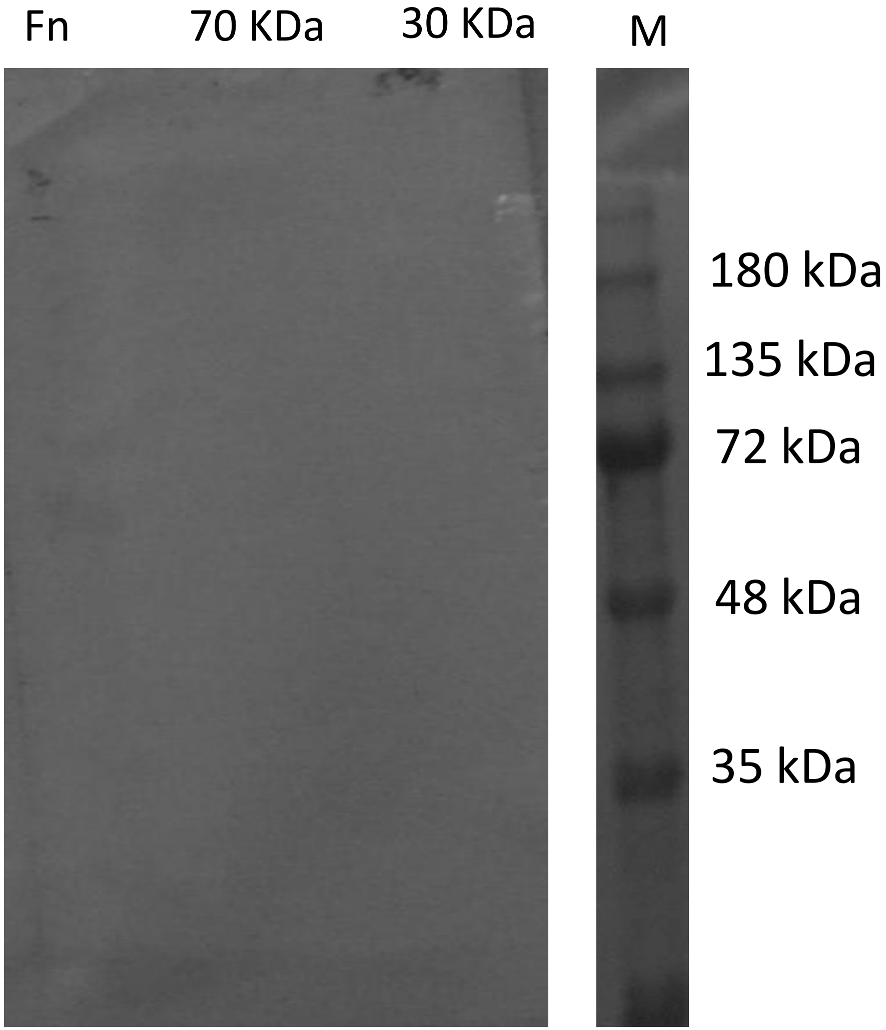

Figure S5

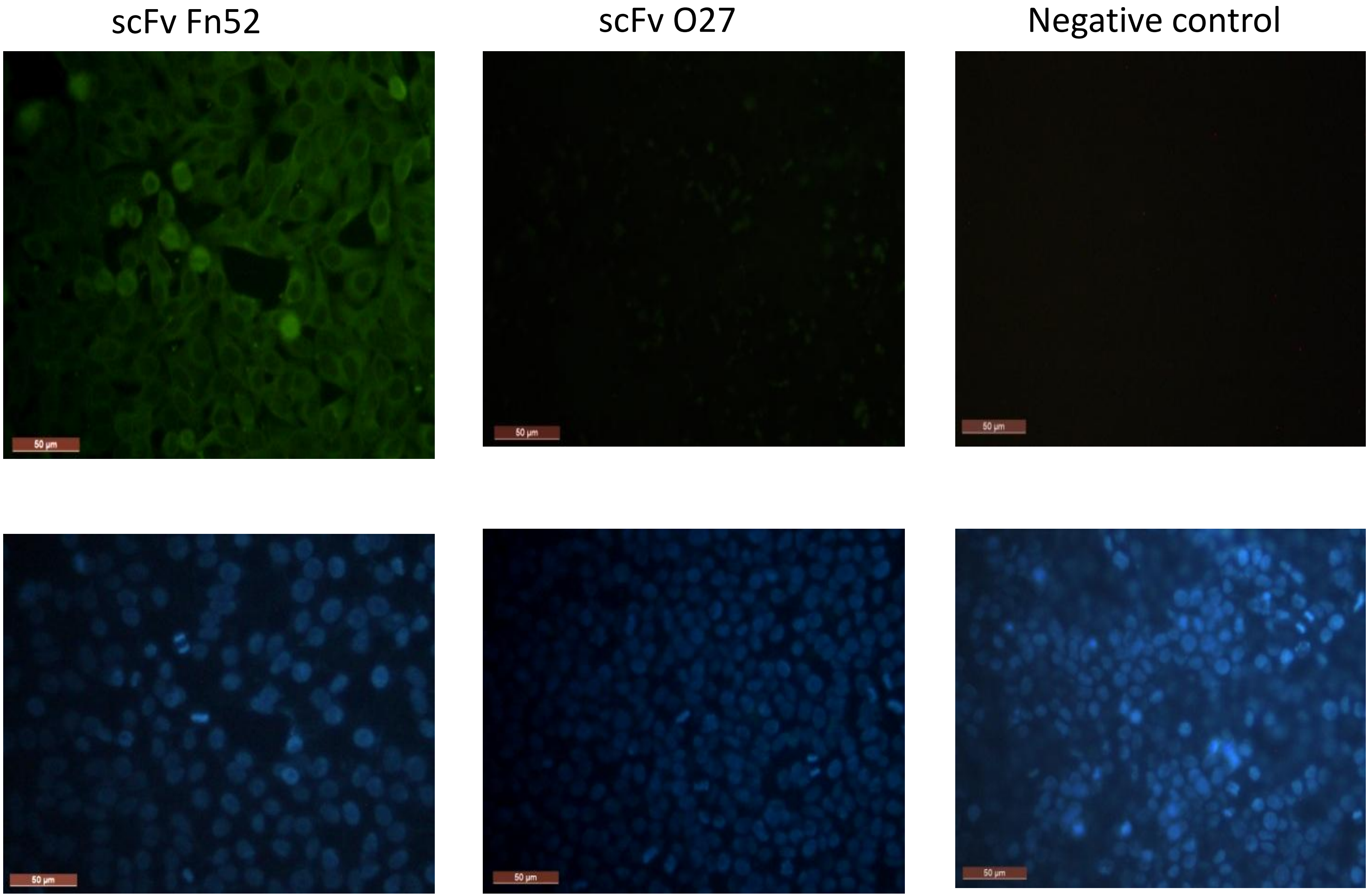

Figure S6

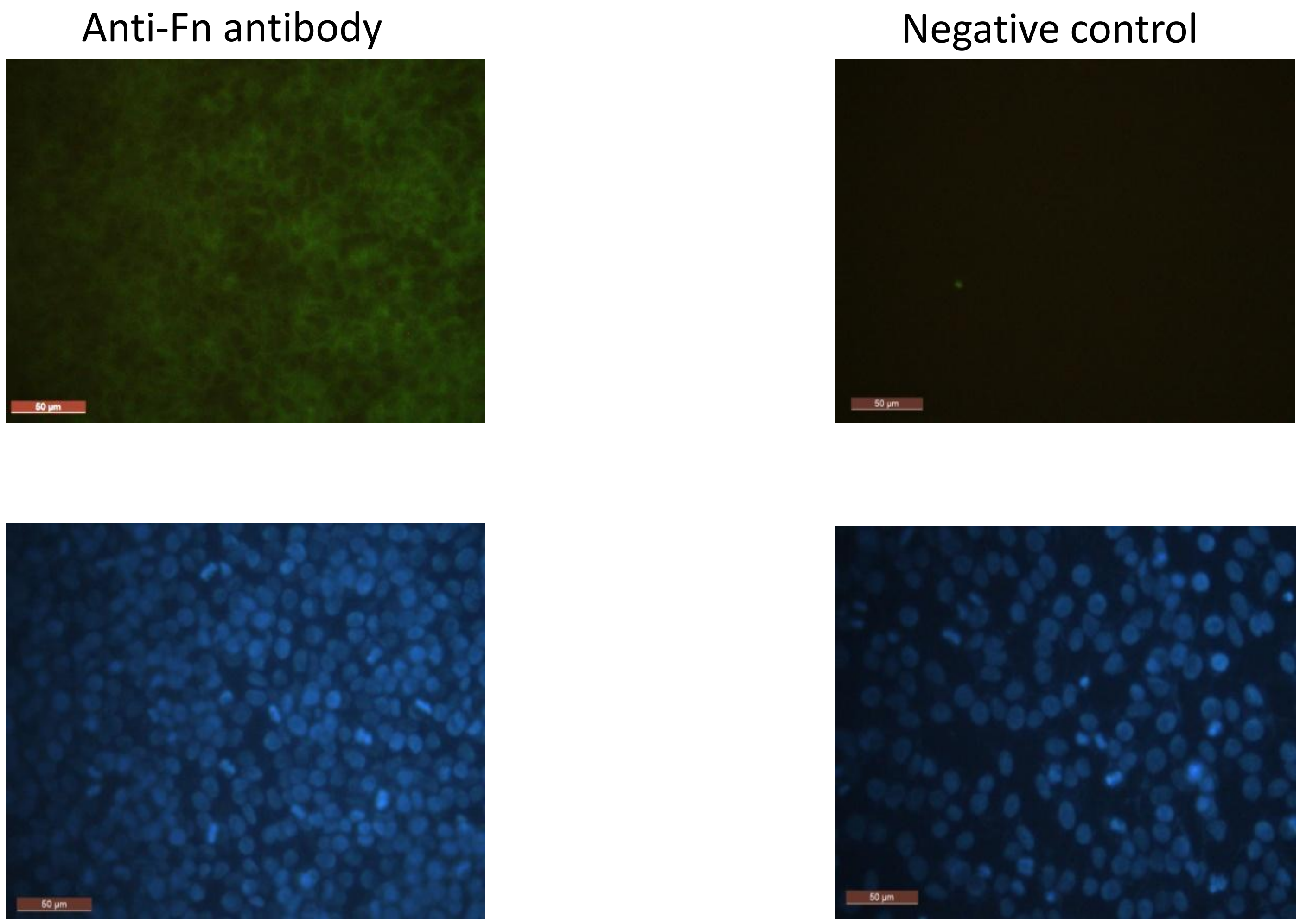

Figure S7

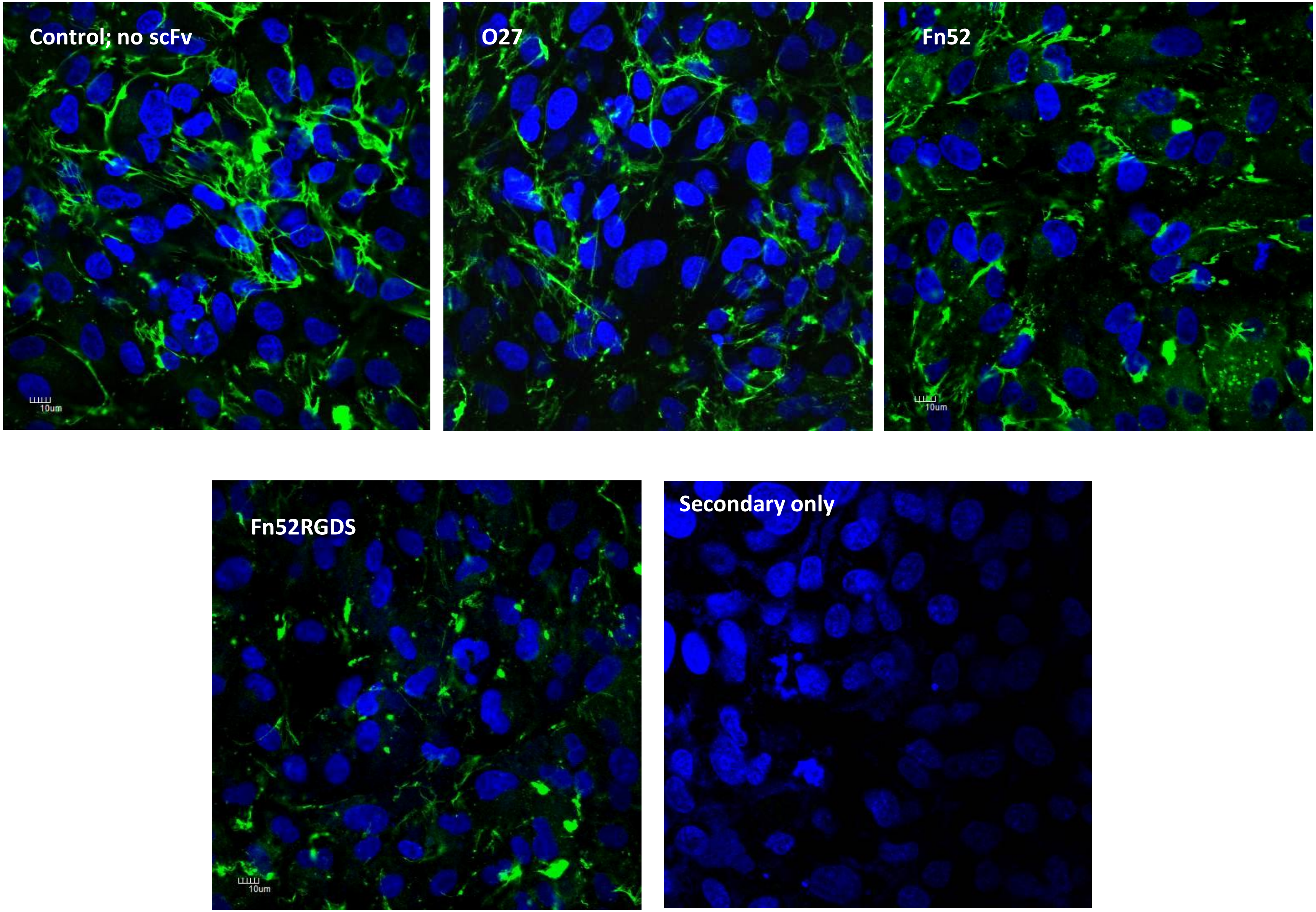
Control; no scFv
10um
O27
Fn52
10um
Fn52RGDS
10um
Secondary only
